# Allergic Bronchopulmonary Aspergillosis with an Atypical Mass-Like Presentation

**DOI:** 10.1155/2022/3627202

**Published:** 2022-06-13

**Authors:** Mahmoud Ibrahim Mahmoud, Alaeldin Elfaki, Ziad A. Alhaj, Abir Hamad Said

**Affiliations:** Imam Abdulrahman Bin Faisal University, King Fahd Hospital of the University, Respiratory Medicine Unit, Al Khobar, Saudi Arabia

## Abstract

Allergic bronchopulmonary aspergillosis is an uncommon condition characterized by airway hypersensitivity to Aspergillus fumigatus, resulting in worsening asthma control and bronchiectasis progression. It is associated with various radiological features. Here, we describe a 53-year-old lady with atypical CT chest finding as soft tissue density masses in both lungs evaluated initially as a lung tumour. The diagnosis was particularly challenging given the history of undiagnosed asthma. Nevertheless, bronchoscopy findings of mucus impaction and blood eosinophilia redirect the clinical thinking toward ABPA. Laboratory examination showed elevated total IgE, Aspergillus fumigatus IgE, and Aspergillus niger IgE. Shortly after treatments with systemic steroids, our patient showed a symptomatic improvement. Moreover, subsequent follow-up showed a resolution of the radiological opacities.

## 1. Introduction

Allergic bronchopulmonary aspergillosis (ABPA) is a respiratory disorder characterized by hypersensitivity to Aspergillus fumigatus (A. fumigatus) and manifested with poorly controlled asthma, recurrent lung infiltrates, and bronchiectasis [1]. It is seen in patients with bronchial asthma or cystic fibrosis (CF) and occasionally other atopic diseases [2]. ABPA may remain underdiagnosed in many countries over the globe and in 30% misdiagnosed as pulmonary tuberculosis in developing countries [3]. Aspergillus sensitization is characterized by immediate skin hypersensitivity or elevated IgE level A. fumigatus antigen [4]. ABPA is considered a late consequence of aspergillus sensitization. Allergic bronchopulmonary mycosis (ABPM) is ABPA-like syndrome attributed to fungi other than A. fumigatus [5]. Severe asthma with fungal sensitization (SAFS) has recently been described as severe asthma and fungal sensitization approaching ABPA without bronchiectasis or mucous plugging and IgE level less than 1000 IU/ml [6, 7]. The prevalence of ABPA is estimated about 1-2% of patients with asthma and 1-15% of patients with CF [8, 9]. There is limited data about ABPA in Saudi Arabia, which suggests that ABPA may be underdiagnosed and associated with other species [10]. The estimated prevalence of ABPM in Saudi Arabia is about 2.7%, predominant in females, with Aspergillus niger (A. niger) being the more common species [11].

## 2. Case Report

This 53-year lifelong nonsmoker lady presented with acute onset pleuritic chest pain, and shortness of breath started a few hours before hospital attendance. The patient denies cough, sputum production, haemoptysis, or other respiratory symptoms at the time of presentation. There were no constitutional symptoms, namely, loss of weight, poor appetite, fever, or excess sweating. Importantly, no family history of asthma, malignancy, or venous thrombosis was noted. She does not keep pets at home.

Clinical examination at the A/E showed normal vital signs apart from a respiratory rate of 20 cycles per minute. There were reduced chest expansion and reduced breath sound on chest examination. Other system examinations were unremarkable.

**Figure 1:**
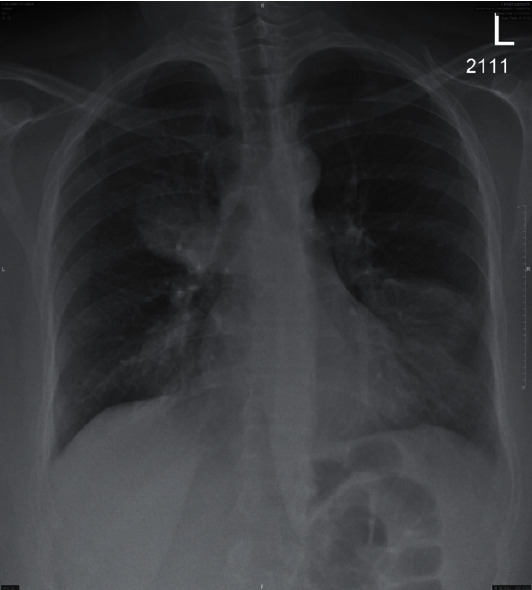
CXR showed bilateral opacities over the right middle and left lower zones.

**Figure 2:**
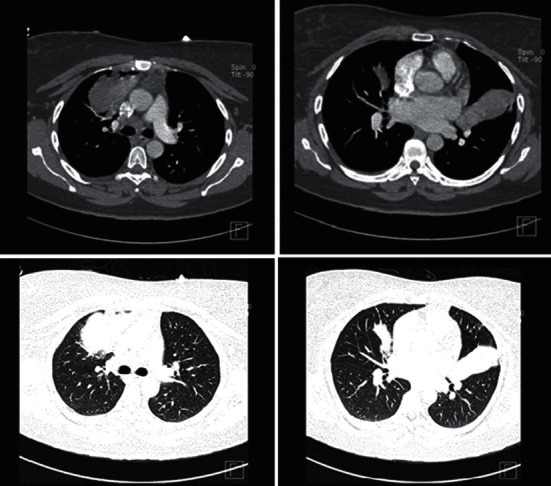
Computed tomography pulmonary angiography shows bilateral mass-like density with finger in glove sign.

Laboratory tests showed peripheral eosinophilia with absolute count 1.0 K/*μ*l (0.8) representing 16%, elevated CRP of 13 mg/dl, and ESR of 60 mm/hr. Her CXR showed bilateral opacities over the right middle and left lower zones (Figure 1). The D-dimer was also raised (0.72 *μ*g/ml FEU). Because of acute presentation of pleuritic chest pain with shortness of breath and elevated D-dimer, the team requested CTPA, which was negative for pulmonary embolism but showed soft tissue density masses in both the right and left lungs (Figure 2).

Initially, she was managed with intravenous levofloxacin, bronchodilator, analgesia, and prophylactic anticoagulation by A/E colleges and later referred to our respiratory team as a case of possible lung mass for bronchoscopy.

On further questions, she gave a previous history of intermittent shortness of breath with a wheezy chest triggered by dust and smoke. Moreover, she had a medical review twice and responded to nebulized bronchodilators. Yet, she was never prescribed long-term inhalers.

**Figure 3:**
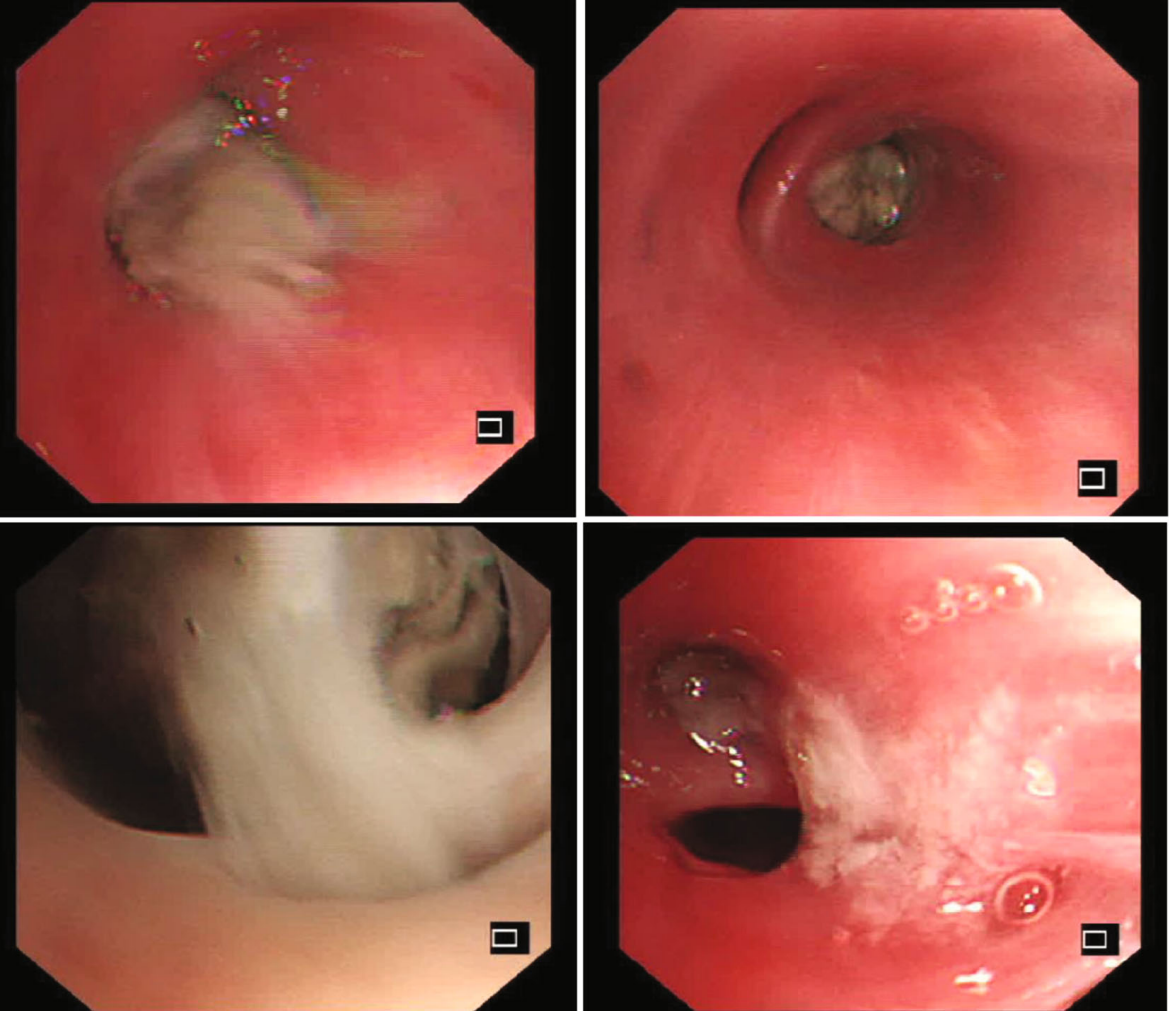
Flexible bronchoscopy revealed whitish, cheesy, and very viscid mucous plugging, which was challenging to be removed by suction.

Flexible bronchoscopy was performed, which illustrated a whitish, cheesy, and very viscid mucous plug. It was challenging when attempting suction (Figure 3). The eosinophil count was 9%, and segmented neutrophils were 68% on bronchoalveolar lavage (BAL). The BAL cultures grew Streptococcus pneumoniae and Aspergillus species (not determined). Other tests were negative, including cytology and acid-fast bacilli stain and cultures.

In view of asthma-like symptoms, peripheral eosinophilia, radiological changes, and mucous plug, allergic bronchopulmonary aspergillosis was suspected. Therefore, we requested total IgE level and Aspergillus-specific IgE. Empirical treatment with methylprednisolone 40 mg daily was initiated for presumable diagnosis of ABPA, awaiting laboratory confirmation.

Later, results came with a total IgE of more than 5000 IU/ml, Aspergillus fumigatus IgE of 19.6 kU/l (elevated), and Aspergillus niger IgE of 52.8 kU/l (elevated).

We discharged her on oral prednisolone 40 mg daily and a plan to taper down gradually in addition to asthma management with a salbutamol inhaler when needed (budesonide/formoterol 160/4.5 mcg turbuhaler twice daily and montelukast 10 mg tablet once daily).

Two weeks later, her symptoms resolved, and a chest X-ray showed the resolution of previously seen bilateral opacities.

## 3. Discussion

In 2013, the ISHAM working group established their criteria to diagnose ABPA. It consists of predisposing conditions (asthma or CF), mandatory criteria, and other criteria [1]. The mandatory criteria require an elevated total IgE level of more than 1000 IU/ml in addition to the presence of either an immediate cutaneous reaction to A. fumigatus or a high level of specific IgE against A. fumigatus [1]. The other criteria are three points; two of three must be present. These are IgG antibodies against A. fumigatus, pulmonary opacities on chest radiography, and eosinophil more than 500 cells/*μ*l in a patient not taking steroids [1].

Generally, patients present with poorly controlled asthma, productive cough, and haemoptysis [12]. Analysis of 113 patients with ABPA [13] found that cough and shortness of breath (99%) were the commonest symptoms, fever was in 80%, nasal symptoms were in 45%, haemoptysis was in 41%, and mucus plug was seen in 37%. In a retrospective review of 77 patients diagnosed with ABPA, chest pain was found in 9% of patients, weight loss in 30%, and night sweats in 12%, and 44 patients (58%) were misdiagnosed with tuberculosis, pneumonia, lung abscess, or lung cancer [14]. Eosinophilic pleural effusion was reported in a case presented with cough and pleuritic chest pain [15]. The bronchopleural fistula was also evident in patients with complicated ABPA who present with fever and chest pain [16].

Radiological findings are widely variable, and normal chest radiology does not exclude ABPA; the radiographic findings include multilobar central bronchiectasis, consolidation, mucous impaction, lobar or segmental collapse, centrilobular nodules, and mosaic attenuation [17]. Although chest CT findings are useful in diagnosing ABPA, few studies have reported CT chest findings in patients with ABPA. Patients with ABPA can present with normal CT of the chest. Central bronchiectasis cannot be considered a characteristic feature as peripheral bronchiectasis is commonly observed. Consolidation is an uncommon finding of ABPA, while high-attenuation mucus, if present, is a characteristic finding of ABPA [17]. The presentation of ABPA as large pulmonary masses is distinctly uncommon. These masses are usually attributed to mucus plugging of bronchi and distal accumulation of secretions [18]. ABPA with mass-like presentation can easily be confused with lung cancer as reported cases in which PET scans, done for staging and treatment planning, were positive [19]. Kaur and Sudan [20] have found central bronchiectasis (CB) evident in 78% of patients with ABPA and concluded that CB combined with centrilobular nodules and HAM is strongly suggestive of ABPA.

ABPA could be classified as ABPA-S (serologic), ABPA-CB (central bronchiectasis), and ABPACB-HAM (central bronchiectasis-high attenuation mucus). Based on the presence or absence of HAM, it should be graded as ABPA-S (mild), ABPA-CB (moderate), and ABPA-CB-HAM (severe) [21].

Bronchoscopy may show bronchial impaction with mucous plug [22] as in our patient. Series of cases of ABPA in CF with mucous plugs had been treated successfully with sequential bronchoscopy using the installation of recombinant human DNase to relieve lobar collapse [23]. The current diagnostic criteria of ABPM do not highlight the role of bronchoscopy and pathological examination. However, bronchoscopy and pathological examination still have fundamental importance for some atypical patients with suspected ABPM. Clue for the diagnosis of ABPA includes brown mucus in the bronchus and a considerable amount of eosinophil infiltration in bronchial mucosa biopsy. However, the chance of finding fungus is not high in tissue sections except in large pneumonectomy specimens [24].

ABPA has been divided into five stages [25]: (1) acute: the patient is diagnosed with ABPA with typical features; (2) remission: the patient has no symptoms, no new radiological infiltrate, and no increase in total IgE for at least six months; (3) exacerbation: manifested by new pulmonary infiltrates, peripheral eosinophilia and IgE level double the remission level; (4) steroid dependence: the patient becomes unable to taper off the steroid; and (5) fibrotic lung disease: chest imaging shows irreversible fibrosis.

The mainstay of ABPA management is to suppress the immune response by using anti-inflammatory agents and reduce the fungal burden by using antifungal agents [26]. Although the dose and duration of treatment were unclear, oral glucocorticoids are currently the preferred treatment in the management of ABPA [27]. There are two different glucocorticoid dosing protocols in ABPA: high-dose (0.75 mg/kg/day for 6 wk taper by 5 mg every 6 wk; total duration: 8-10 months) and low-dose (0.5 mg/kg/day for 2 wk, taper by 5 mg every 2 wk; total duration: 3-5 months) prednisolone [28]. Yet, low-dose glucocorticoids are as effective as high-dose ones and are associated with lesser side effects [29].

Antifungal therapy based on currently available evidence depends on patient-specific risks, with the following indications; facilitate reduction or tapering of steroids when further exacerbation developed [30]. Oral first-generation triazole, i.e. itraconazole, is effective against A. fumigatus and is predominantly first-line therapy in managing ABPA [31].

In some cases where the steroid dose cannot be reduced or side effects develop, monoclonal agents may be added to the treatment [32]. Omalizumab usage in 16 patients having bronchial asthma with ABPA has shown a significant reduction in steroid dose and exacerbation frequency [33].

Using inhaled corticosteroid (ICS) alone in the management of ABPA showed significant deterioration of symptoms and rise of IgE levels; therefore, using them alone is not recommended [34].

The initial clinical impression in our case is primary lung cancer, based on the CT finding of soft tissue density mass; accordingly, bronchoscopic evaluation was conducted digging for endobronchial tumours. However, bronchoscopic examination instead reveals mucus impaction representative of an inflammatory process. Retrospectively, a review of the soft tissue density morphology seen in the CT scan revealed a finger-in-gloves pattern, particularly on the left lung mass.

Nevertheless, the mass density is not in keeping with high attenuation mucus (pathognomy for ABPA), in which the mucus is denser than the paraspinal skeletal muscle. The combination of bronchoscopy findings and finger in gloves noted on a CT scan with significant eosinophilia noted in CBC direct the clinical suspicion toward ABPA.

Our case is fulfilling the ISHAM diagnostic criteria of ABPA. The obligatory criteria are the total IgE of 5000 IU/ml and Aspergillus fumigatus IgE of 19.6 kU/l, and the two supportive criteria are peripheral eosinophilia with absolute count 1.0 K/*μ*l and the typical radiological features of ABPA. Despite the absence of documented diagnosis of bronchial asthma, we believe that her background symptoms of intermittent shortness of breath and wheeze that responded to a bronchodilator are suggestive of undiagnosed bronchial asthma.

Therefore, our patient was staged as an acute ABPA-CB (central bronchiectasis); consequently, a low-dose steroid regimen without an antifungal agent was started. Our patient showed clinical and radiological improvement a few weeks after starting the treatment.

## 4. Conclusion

In conclusion, ABPA can present as a soft tissue mass that is mistaken as a lung tumour. Although current diagnostic criteria do not highlight bronchoscopy's role in the diagnosis, we believe bronchoscopy findings and blood eosinophilia play a fundamental role in an atypical patient, as in our case. Importantly, careful medical history, particularly regarding undiagnosed bronchial asthma, is helpful for diagnosing ABPA. Here, we found airway hypersensitization to both A. fumigatus and A. niger which is the commonest causative agent in Saudi Arabia. Further studies are needed to determine whether this atypical combination of allergy to both A. fumigatus and A. niger could explain the atypical radiological presentation.

## Data Availability

The data used to support the findings of this study are included within the article.
